# Balancing between predation risk and food by boreal breeding ducks

**DOI:** 10.1002/ece3.11011

**Published:** 2024-02-09

**Authors:** Sari Holopainen, Elmo Miettinen, Veli‐Matti Väänänen, Petri Nummi, Hannu Pöysä

**Affiliations:** ^1^ Department of Forest Sciences University of Helsinki Helsinki Finland; ^2^ Organismal and Evolutionary Biology Research Programme, Faculty of Biological and Environmental Sciences University of Helsinki Helsinki Finland; ^3^ Department of Environmental and Biological Sciences University of Eastern Finland Joensuu Finland

**Keywords:** alien predator, camera trap, invertebrate, nest predation, waterbird, wetland

## Abstract

Wetlands belong to the globally most threatened habitats, and organisms depending on them are of conservation concern. Wetland destruction and quality loss may affect negatively also boreal breeding ducks in which habitat selection often needs balancing between important determinants of habitat suitability. In Finland duck population trajectories are habitat‐specific, while the reasons behind are poorly understood. In this research, we studied the balance of nest predation risk and invertebrate food abundance in boreal breeding ducks in Finland at 45 lakes and ponds in 2017 and 2018. We surveyed duck pairs and broods from these and 18 additional water bodies. We evaluated nest predation by monitoring artificial nests with camera traps over a 7‐day exposure period and sampled invertebrates from water bodies using emergence and activity traps. Camera trap results indicate that predation risk was higher in the water bodies surrounded by agricultural land than in forestland. Ponds (seasonal, beaver, and man‐made) had lower nest predation risk, and they were also more invertebrate‐rich than permanent lakes. In addition, artificial nests further away from water bodies had higher survival than shoreline nests. Habitat use of duck pairs was not associated with invertebrate food, but duck broods preferred habitats rich in food. High nest predation pressure in shorelines of especially agricultural landscapes may contribute to the declining population trends of ducks in Finland. Controlling predators could be an important conservation action to improve duck breeding success. This research underlines the benefits of the availability of different water body types for breeding ducks. There is an urgent need to pay attention to protecting seasonal ponds, while the lack of flooded waters may be mitigated by favouring beavers or creating man‐made ponds.

## Graphical abstract

We studied the effects of wetland quality on boreal breeding ducks from two important aspects: nest predation and food availability. Duck food availability is low and nest predation pressure is high at permanent lakes, while ponds (seasonal, beaver, man‐made) were shown to be food‐rich habitats with low nest predation pressure. Our results emphasize the benefits of different wetland types in the landscape for the breeding ducks.

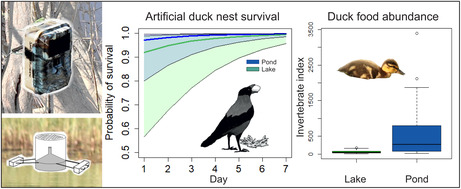

## 1 INTRODUCTION

Humans have altered wetland ecosystems in numerous ways across the globe, especially through drainage for agricultural land (Davidson, 2014; Gibbs, 2000; Hu et al., 2017; Kingsford et al., 2016) and to increase wood production (Kuusisto et al., 1998). In addition to direct wetland destruction, climate warming might affect wetland formation and hydroperiods due to decreased precipitation, increased evapotranspiration and permafrost loss (Chapin et al., 2010; McMenamin et al., 2008; Riordan et al., 2006). In Eurasia, over‐exploitation of wetland‐creating beavers has affected the amount and dynamics of wetlands (Halley et al., 2021). Furthermore, wetland quality is threatened, for instance, due to agricultural and forestry‐driven eutrophication and brownification, in addition to alien species introductions (Fox et al., 2020; Guillemain et al., 2013; Holopainen & Lehikoinen, 2021; Ma et al., 2010; Nummi, Väänänen, et al., 2019; Ramsar Convention Secretariat, 2010). All in all, human‐induced environmental wetland change has therefore impacted aquatic animals at multiple levels of organization from individuals to landscapes (Sievers et al., 2018).

Habitat characteristics are important determinants of breeding densities and production of ducks on boreal wetlands (Holopainen et al., 2015). Many boreal lakes lack the habitat structure (i.e. shallow shores profitable for duckling foraging) and sufficient food resources (i.e. invertebrate production) to support breeding ducks, making them unsuitable for brood rearing (Sjöberg et al., 2000). Indeed, all lakes used by duck pairs are not suitable for broods due to food limitations (Gunnarsson et al., 2004; Sjöberg et al., 2000). Duckling mortality at lakes with limited food resources, in particular invertebrates, is high (Gunnarsson et al., 2004; Nummi & Hahtola, 2008). In spring, however, patterns of snowmelt create annual variation in the nature and extent of shallow flooded lakeshores, affecting littoral ecosystem productivity (Larmola et al., 2004). These seasonal floods, in addition to seasonal ponds, commonly dry during the summer but offer important food‐rich habitats for duck broods in early summer (Holopainen et al., 2014). In addition, habitat engineering by beavers (*Castor* spp.) modifies oligotrophic, sharp‐edged boreal lakes into productive shallow wetlands with ambiguous shorelines. Both beaver ponds and seasonal ponds typically have varying shorelines and possibly no fish or low fish densities (Nummi & Hahtola, 2008).

Habitat selection of breeding ducks is not straightforward but will possibly lead to trade‐off situations both between and within different stages of the breeding season. For example, experimental data by Gunnarsson and Elmberg (2008) suggests a trade‐off between wetland use and nest survival in forested versus agricultural landscapes in the mallard (*Anas platyrhynchos*). The results showed that wild waterfowl, including mallard, seemed to prefer agricultural landscapes while facing higher nest predation risk there. While predation risk largely determines nest site use and nesting success (Holopainen et al., 2015; Jaatinen et al., 2022), food resources and habitat structure are the key characteristics affecting habitat use by duck pairs and broods as well as subsequent breeding success in boreal lakes (Holopainen et al., 2015). At wetlands, complex habitat structure and luxuriant vegetation are linked, as the abundance of emergent vegetation typically increases from nutrient‐poor oligotrophic to nutrient‐rich eutrophic lakes (Holopainen & Lehikoinen, 2021; Kauppinen & Väisänen, 1993; Nummi & Pöysä, 1993).

Changes in important boreal environmental characteristics may already have affected breeding ducks negatively. Finnish national duck pair surveys show declining trends for several species, but those breeding in eutrophic lakes have declined more than in oligotrophic lakes (Holopainen et al., 2024; Lehikoinen et al., 2016; Pöysä et al., 2013). In addition to detrimental effects of vegetation overgrowth and water quality changes at eutrophic waters (driven for instance by agriculture and forestry; Holopainen & Lehikoinen, 2021), disproportionally increased predator pressure is one of the suspected reasons for the differences in population trajectories between habitats and also between species within habitats (see Holopainen et al., 2024; Pöysä et al., 2019; Pöysä & Linkola, 2021), potentially impacting flyway‐level trends in population size and structure (e.g. Brides et al., 2017).

Indeed, artificial duck nest experiments in northern Europe have shown that nest predation is high around wetlands in agricultural landscapes (Holopainen et al., 2020a), where alien mammals are increasing in abundance (Pöysä et al., 2023). As a result, ducks nesting along the shorelines of eutrophic lakes have likely experienced an increase in predator diversity and abundance that results in increased nest predation risk, which has contributed to population declines (Holopainen et al., 2021, 2024; Pöysä & Linkola, 2021).

In this article the complex habitat‐based associations with duck breeding success will be analysed. We will assess whether and how habitat use and brood production (broods per pair) by boreal breeding ducks result from a trade‐off between nest predation risk and food availability. Specifically, we used camera trapping at artificial nests (mimicking dabbling duck nests) to measure nest predation risk at both the local habitat (shoreline nests vs. forest nests) and landscape (proportion of agricultural land vs. forest in the landscape) scales. Next, we assessed the habitat use of both breeding pairs and broods emphasizing the role of landscape and food availability (invertebrate abundance). In addition, we measured brood production at the landscape scale. We predict that while eutrophic water bodies in agricultural landscapes produce more invertebrates, they will also have a higher nest predation rate, which translates into lower brood production. Furthermore, contrary to permanent lakes, we predict that flooded ponds offer the most food‐rich brood habitats but also safe nesting places due to fluctuating water levels. These marginal habitats may therefore provide important breeding habitats for boreal ducks.

## 2 METHODS

### 2.1 Study areas

**FIGURE 1:**
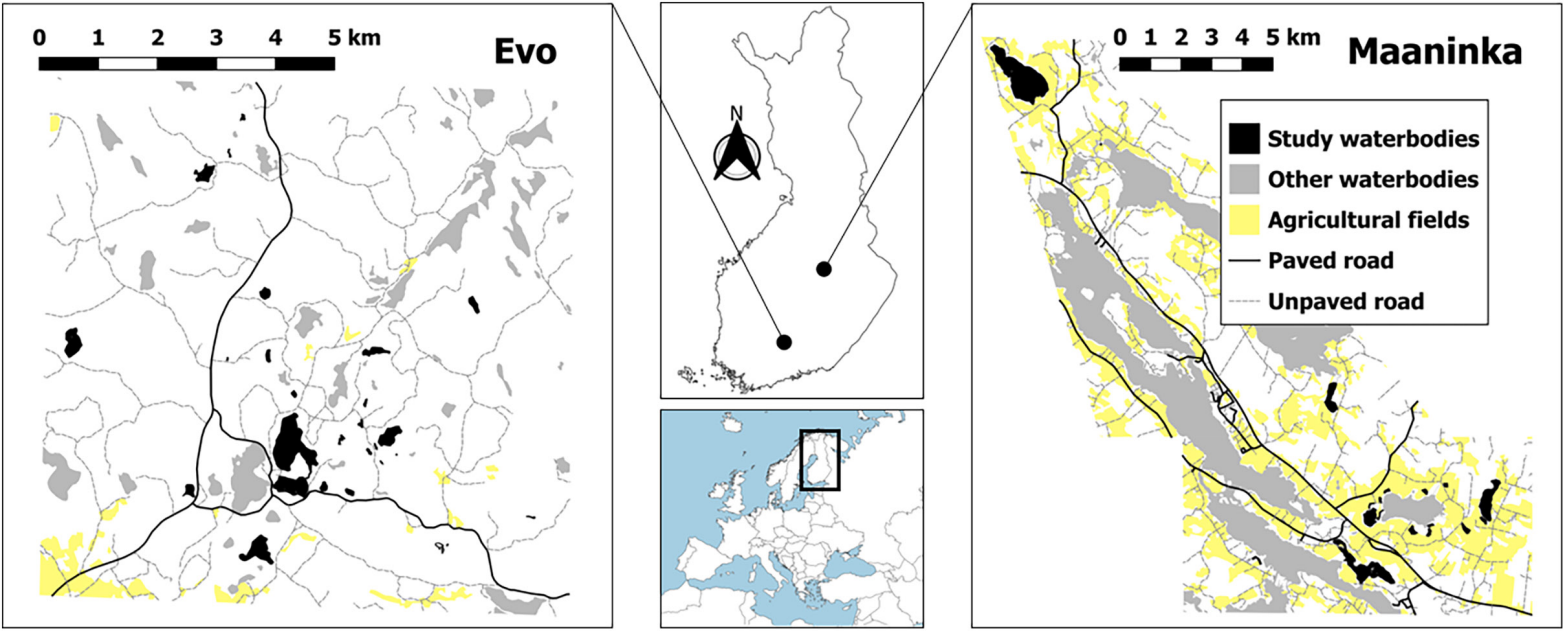
Location of Finland in Europe (panel in the middle) and the maps of the two study areas Evo (panel on the left) and Maaninka (panel on the right). Lakes used for camera trap experiment and invertebrate trapping are indicated in black colour (*Sources*: Esri, National Land Survey).

Our study was conducted at water bodies at two areas in Finland, Evo and Maaninka (Figure 1). Both areas have permanent lakes, which carry water through the summer. Lake shorelines may be affected by spring floods, but otherwise the water level is rather constant. The trophic level among these lakes varies from oligotrophic to eutrophic (see Holopainen & Lehikoinen, 2021). In addition to permanent lakes, in both areas the study included other water bodies, which were shallow and had temporally varying shorelines: seasonal ponds, beaver ponds and man‐made ponds (hereafter ponds). For this study, we selected water bodies surrounded by different proportions of forest versus agricultural land: different landscapes to cover the whole gradient from fully forested to mainly agricultural were chosen. Landscape might affect not only the lake's trophic status but also the nest predator community, as stated by Holopainen et al. (2020a). The same mammalian predators and all common corvid species can potentially occur in both study areas (Lindén et al., 1996; Valkama et al., 2011).

Evo in southern Finland (61°12′ N, 25°07′ E) represents a typical boreal forest landscape. Human settlements are scarce, with few agricultural fields (hereafter fields) inside the study area and larger agricultural lands south of the studied water bodies. In Evo we undertook duck surveys at 45 water bodies (27 permanent lakes, 10 beaver ponds and 8 seasonal ponds) within a c. 39‐km^2^ area. Due to the wildlife camera and time limitation, we chose 9 beaver ponds, 8 seasonal ponds and 12 permanent lakes from the 45 study water bodies for nest predation experiments and invertebrate surveys (beaver pond and seasonal pond occurrence was evaluated annually and only flooded ones were included to the experiment). Water body size for permanent lakes varied between 0.7 and 37.6 ha (median 4.3 ha, standard deviation [SD] = 10.4) and shoreline length between 0.3 and 3.7 km (median 0.9 km, SD = 1.0). Water body size for ponds varied between 0.04 and 6.4 ha (median 0.4 ha, SD = 1.6) and shoreline length between 0.07 and 1.6 km (median 0.4 km, SD = 0.4).

Maaninka in eastern Finland (63°15′ N, 27°30′ E) is a mosaic of agricultural land and forests with some internationally important bird‐lakes (Natura 2000 and IBA‐lakes; Leivo et al., 2002). The area represents typical agricultural landscape of Finnish lake district. We made duck surveys at 18 water bodies across c. 47 km^2^: 17 water bodies were used for the experiments, among them the important bird‐lakes. We included all seasonal ponds that occurred during the study years and to which we had permission granted from the landowners. In total there were six permanent lakes, two man‐made ponds and nine seasonal ponds for nest experiments and invertebrate surveys. Water body size for permanent lakes varied between 6.2 and 148.7 ha (median 30 ha, SD = 52.9) and shoreline length between 1.7 and 7.8 km (median 3.1 km, SD = 2.6). Water body size for ponds varied between 1.6 and 4.3 ha (median 2.4 ha, SD = 1.0) and shoreline length between 0.5 and 1.8 km (median 1.0 km, SD = 0.4).

### 2.2 Duck surveys

The duck species studied here are ground nesting and distributed widely in the boreal zone: mallard, common teal (*Anas crecca*; hereafter teal), Eurasian wigeon (*Mareca penelope*), northern pintail (*Anas acuta*), northern shoveler (*Spatula clypeata*), garganey (*Spatula querquedula*) and tufted duck (*Aythya fuligula*). All were observed to breed in Maaninka, but only mallard and teal bred at Evo. We conducted duck pair and brood surveys in 2017 and 2018 using the standard waterbird round count method (Koskimies & Väisänen, 1991). In the round count, we surveyed the water bodies by moving around the lake by a boat, stand‐up paddling board or by foot near the shoreline so that all the settled birds were detected with a high probability. Detection probability has not been tested specifically for the round count method. However, because pair surveys are done before the vegetation has started to grow (good visibility) and birds hiding in the sparse, old vegetation typically respond to the observer by taking flight, we assumed detection was high, especially considering that the water bodies studied were relatively small (see also Koskimies & Pöysä, 1989). When it comes to brood surveys, detection probability is assumed to be higher in the round count method than in the point count method (e.g. Pöysä, 1989), the latter being a widely used alternative waterbird survey method in Finland (Koskimies & Väisänen, 1991).

We conducted pair surveys in April and May right after the ice melt, when duck pairs occupy their breeding wetlands and are preparing for nesting. Ice melting sets an exact time frame for the duck surveys (Pöysä, 1996, 2019), making it possible to calibrate the phenology between different areas and years (at Evo ice melts c. 2 weeks earlier than in Maaninka and we took this into account when timing pair surveys and nest experiments within each study area). We considered pairs and lone males as pairs following the standard protocol by Koskimies and Väisänen (1991). Also groups of 2–4 males were used to estimate the number of pairs (i.e., 2–4 pairs). If the number of females surpassed the number of males at a wetland, the number of females was used instead. We conducted brood surveys twice a year in early June and July and recorded the species, number and age of ducklings for each brood (Pirkola & Högmander, 1974). When studying brood habitat use, we used all brood observations for the analyses to determine the diverging habitat use of different age classes (i.e. some broods might occur twice in the analyses). When analysing brood production and density, we identified every brood based on their age and count, thus counting each brood once (i.e. assuming they did not change sites between the surveys).

### 2.3 Artificial nest survival experiment with camera trapping

We conducted artificial nest experiments with wildlife camera traps in 2017 and 2018 to study nest predation rates at the water bodies. The nest experiment was started during the pair survey, right after ice melt (Evo before Maaninka, see Section 2.2), the time when ducks initiate egg laying. One nest experiment round took 7 days, and all the nests of a round were established and deconstructed on the same day between 9 am and 4 pm. We carried out two rounds of nest experiments with different sets of water bodies in each study area (i.e. two 1‐week experiments with 20–24 nests at the time).

We placed nests in sites where a ground‐nesting duck hen could possibly lay a clutch, based on our own experience (nest site selection of boreal ducks is poorly studied, review by Holopainen et al., 2015; see also Väänänen et al., 2016). Some duck species nest at the shoreline, while others can place nests in the forest far from water bodies (even 1 km away based on our own observations), so the artificial nest sites reflected this distribution.

Each nest contained two farmed mallard eggs and some down (from legally harvested wild mallard females), mimicking the situation in the early stage of egg laying. We constructed nests to resemble real ones: natural nest material from the nest surroundings were collected to form c. 20 cm wide nest cup and cover the eggs lightly. We did not cover eggs with down since ducks do not typically cover them before starting incubation. We set nests under small trees or bushes, so they were hardly detectable from above. In open areas we established nests within tussocks. We avoided making trails to the nests, while only one visit per site would not typically leave a trail in the boreal landscape. We used rubber gloves to construct nests, and cameras were attached with iron wire instead of nylon straps to minimize human scent. We did not visit nests during the 7‐day exposure period.

We established experimental nests in pairs around the water bodies: we placed shoreline nests less than 5 m from shorelines and forest nests at least 70 m (range 70–1400 m) from the shoreline nest to the nearest forest. At Evo, every water body had only one nest pair a year, while at Maaninka there were fewer but larger water bodies, and thus 2–3 nest pairs around four water bodies were established. The same nest sites were used in both years to minimize the site effect. At Evo (mostly government‐owned forest) shoreline nest sites were randomly selected from eight sectors around the water bodies. Those sectors that we choose had to have at least 140 m distance to other lakes: forest nests had to be at least 70 m away from any water body shoreline. If this was not possible, the nests were established in the adjacent sector. We avoided placement close to streams, clear cuttings and roads, because they could affect predator movements. Forest nests in Evo situated 70–90 m away from the water body shoreline nest. At Maaninka most land is private, so access was limited due to land‐use permissions. Water bodies are typically surrounded by arable fields; here the average forest nest distance from the shoreline nest was 650 m (range 70–1400 m).

In 2017 there were 46 nests at Evo and 42 at Maaninka, and in 2018 the numbers were 48 and 42, respectively (in total 178 nests; from which one nest was accidentally destroyed by the landowner in 2018). The density of the experimental nests was c. 1.2 nest/km^2^ at Evo and 0.9 nests/km^2^ at Maaninka. We measured nest density by finding the outermost points of the study areas: we made 500 m buffers for the nests and used these buffers to define the outermost borderlines. In Evo, where the nests were evenly distributed within the area, we only had one framing to measure the density. In Maaninka we had two separate sub‐areas more than 10 km apart.

We set wildlife cameras (20 Uovision UV595‐Full HD 12 MP and four Niteforce Professional Trail Camera 12 MP; MP = megapixel) at artificial nests to identify predator nest visits and depredation time. Cameras were active the whole 7‐day period responding to movement and were adjusted to take three pictures in a row, followed by a 1‐min pause. Light‐triggered passive wildlife cameras were ca. 1–1.5 m from nests, attached on trees or 1 m stakes. We used short distance because it increases the observation possibility of the nest visitors (Randler & Kalb, 2018), and we were also able to measure fate of the eggs from the pictures (see e.g. Holopainen et al., 2020b).

We compared the daily survival of forest versus shoreline nests for predation risk, based on 175 nests (88 forest and 87 shoreline nests that survived the entire study period or with the known depredation time [68 nests depredated]) surrounding in total of 46 water bodies. Furthermore, we compared the daily survival of shoreline nests around permanent lakes and ponds based on 87 nests (41 with exact depredation time).

### 2.4 Invertebrate surveys

We conducted invertebrate trapping in the water bodies in June 2017 and 2018 during the first brood survey. All details of the trapping procedure were identical between the 45 studied water bodies (one seasonal pond used for camera trapping drained before invertebrate trapping). We trapped free‐swimming aquatic invertebrates with the activity trap described in Elmberg et al. (1992). We used 1‐L glass jars with transparent plastic funnels (with 100‐mm openings at the large end and 20‐mm ones at the narrow end) suspended in the middle of the water column within the reach of the ducks (c. 25 cm from the water surface) as close to the shoreline as possible. We used 1 mm sieves to collect the samples, and the catch was analysed in the laboratory by using microscopes. We captured emerging insects with emergence traps similar to those described by Danell and Sjöberg (1977). We used white 5‐L plastic buckets with plastic funnels (with 200‐mm openings at the large end and 40‐mm openings at the narrow end). The emergence traps floated at fixed sites upon two styrofoam panels (c. 30 × 6 × 4 cm) attached to the bucket with metal rods so that about 5 cm of the trap rested below the water surface. The bottom of the bucket (i.e. on the top of the trap) was replaced with a white net: the net lets the light through making it possible for the invertebrates to head up to the gauze bags inside the buckets.

We trapped all the water bodies in each area for 1 week. We set three traps of both types per water body for 48 h at fixed sites on the shore so that seemingly the best shore section with wide and high vegetation and the poorest shore section with a narrow or non‐existent vegetation belt were sampled in each water body (Suhonen et al., 2011). In addition we set one trap in the average vegetation.

We identified all trapped invertebrates and assigned their size according to the taxon list and length categories provided by Nudds and Bowlby (1984). In some cases, however, the prey animals within a given taxon did not fit those length categories, and we used an appropriate length category instead. Because the size of the species caught varies considerably and different‐sized species dominate in different lakes, we multiplied the number of individuals within each taxon by the mean size of the appropriate length category. Thus, our invertebrate index is expressed as ‘millimetres’ of invertebrates: this can be used as a reflection of the energetic content of the invertebrate food. The invertebrate index is an average of three traps, since in a few cases it was not possible to get samples due to, for example fallen traps or detached funnels (1 activity trap in 2017, 2 emerging and 2 activity traps in 2018). We combined the invertebrate measures from the two trap types to give a water body‐level food abundance index (Holopainen et al., 2014) as an index of habitat quality (for more information about local invertebrate catch and species‐specific duck‐invertebrate associations, see Nummi et al., 2013; Nummi & Väänänen, 2001). For the analyses, we scaled this index so that it would correspond better to the variance of other variables and divided index values by 100.

### 2.5 Statistical methods

As shown by Ellis et al. (2020), patterns of nest predation may not be predictable by habitat characteristics at a single spatial scale. In this study, we used two different scales to explain duck and invertebrate abundances, in addition to studying nest survival within and between the artificial nest pairs. First, we used water body type and nest location (shoreline, forest) to control habitat‐scale effects. Second, to study landscape‐scale effects, we quantified the landscape structure (i.e. the field percentage) within a 1 km radius buffer from the shoreline of water bodies using QGIS 2.18.7 (QGIS Development Team, 2017) and topographic vector map (National Land Survey of Finland, 03/2019). Those seasonal ponds that were not shown on the national map were added by hand, based on our observations in the field. We used the 1 km radius, because habitat‐specific effects disappear with a larger zone (Uusihakala, 2021), and on the other hand, with this framing, there were still differences between the landscapes of different water bodies. We excluded all water bodies from the zones in order to count the field percentage of surrounding land areas. In Evo the lands within these zones consisted on average 1% of fields (range 0%–6%) and in Maaninka on average 59% of fields (range 24%–75%), the remainder being mainly forests.

### 2.6 Pair and brood numbers and habitat use

Since we had two different study areas, we first compared pair and brood densities and brood production between these areas. We used Mann–Whitney *U*‐test for independent samples to compare the overall pair and brood densities of all the studied duck species between Maaninka and Evo. Furthermore, as mallard and teal are generalist species and common in both study areas, hence providing sufficient data, their pair and brood densities and brood production between the two areas were compared separately. We used *G*‐test for goodness‐of‐fit to compare species‐specific proportions of brooded and non‐brooded pairs (i.e. brood production) in 2017 and 2018 at Evo with those at Maaninka.

Second, to study habitat use of ducks, we analysed pair and brood numbers in relation to habitat variables. We made this analysis at the lake level and combined observations of all species. Pair and brood data were zero‐inflated, and when exploring the non‐zero part, there was still overdispersion. We thus used zero‐inflated negative binomial models to explain variation in the number of all pairs and broods at the water body level using glmmTMB (Brooks et al., 2017). All the analyses were done in R 3.4.0 (R Core Team, 2017), and we did the data exploration by following the protocol by Zuur et al. (2010). We controlled water body size by including shoreline length (‘SHORE’) as an explanatory variable in all the models. We used field percent (‘FIELD’) to indicate the type of the surrounding landscape around each water body (1 km buffer around the water body) in every model. The invertebrate index indicating the amount of food (‘FOOD’, continuous) and water body type (‘TYPE’, two levels: lake or pond) was used as water body‐level explanatory variables. In addition, we included pair number (‘PAIRS’) in the models explaining brood numbers. However, as data exploration revealed that pair number and shoreline length were strongly correlated (Pearson correlation *r* > .6), we discarded shoreline length and kept the pair number, because the latter should more directly determine possible broods produced. Due to the nested structure of the data, water body ID (‘WATER BODY_ID’) was entered as a random factor. Year effect was excluded because it failed to improve model fit. We fitted all possible model combinations, including the intercept‐only model. Because of model selection uncertainty (several models within ΔAIC < 2, where Δ = AIC_*i*_ − AIC_min_), we calculated the model‐averaged slopes (*β*‐values) of the variables weighted by the Akaike weights, their unconditional standard errors and 95% unconditional confidence intervals; all models were used (see Burnham & Anderson, 2002).

### 2.7 Nest survival

We used GLMM framework to calculate daily nest survival probability by using modified logistic regression, which incorporates the number of exposure days (seven, each beginning at 12 pm) into the link function (Shaffer, 2004). The logistic exposure method is a modification of logistic regression and maximizes the use of nest survival data by treating each measurement day as a discrete trial. Daily nest fate was analysed as a binary response variable (1 = survived, 0 = depredated). In the forest‐shoreline nest location comparison explanatory variables were ‘DATE’ (continuous: 1–7) and ‘HABITAT’ (factorial: shoreline, forest; explaining differences within nest pairs). We used field percentage (‘FIELD’) around water bodies as a landscape‐level explanatory variable (explaining differences between nest pairs). As we established nests in pairs around the water bodies, one in the shoreline and one further away from the shoreline, nest pair (‘NESTPAIR_ID’) was used as a random factor.

When comparing survival of nests in the shorelines of different water body types, the explanatory variables were ‘DATE’ and ‘TYPE’ (two levels: lake, pond). We used ‘NESTPAIR_ID’ again as a random factor, but this time it only meant shoreline nests. We again used field percentage (‘FIELD’) around water bodies as a landscape‐level explanatory variable. Year effect was found to be negligible during the data exploration and was thus discarded from both analyses.

### 2.8 Invertebrate food abundance

We used linear mixed‐effects modelling (nlme package, Pinheiro et al., 2018) to study whether water body type (‘TYPE’, two levels: lake or pond) affects the invertebrate food abundance index, incorporating shoreline length (‘SHORE’) and field percentage (‘FIELD’) as explanatory variables. We used water body ID (‘WATER BODY_ID’) as a random factor.

## 3 RESULTS

### 3.1 Pair and brood numbers and habitat use

Overall brood, but especially pair densities, were higher in Maaninka than in Evo (for pairs, *N* = 117, *U* = 378, *p* < .001; for broods, *N* = 117, *U* = 863, *p* < .001; Table 1). The same pattern was also observed if only teal densities were considered (pairs *N* = 117, *U* = 495, *p* < .001; broods, *N* = 117, *U* = 986, *p* < .001, Table 1). Mallard pair, but not brood density differed between the areas (pairs *N* = 117, *U* = 884, *p* < .001; broods, *N* = 117, *U* = 1267, *p* = .242, Table 1). However, both mallard and teal per capita brood production was higher in Evo than in Maaninka (mallard *G* = 20.7, df = 3, *p* < .001; teal *G* = 52.2, df = 3, *p* < .001; Table 1).

**TABLE 1:** The average, median and range of pair and brood densities (all duck species, teal, mallard/shoreline km) and brood production of teal and mallard in Evo and Maaninka combining the years 2017–2018.

	Pairs/shoreline km	Broods/shoreline km	Broods/pair
	Average, median (range)	Average, median (range)	
All species			
Evo	0.9, 0.0 (0–9.1)	0.3, 0.0 (0–4.5)	
Maaninka	5.1, 3.8 (0–23.1)	1.1, 0.3 (0–15.2)	
Teal			
Evo	0.4, 0.0 (0–9.1)	0.2, 0.0 (0–4.5)	0.35
Maaninka	2.1, 1.4 (0–10.9)	0.5, 0.0 (0–4.5)	0.13
Mallard			
Evo	0.5, 0.0 (0–6.8)	0.1, 0.0 (0–2.3)	0.26
Maaninka	1.5, 0.6 (0–7.1)	0.2, 0.0 (0–1.7)	0.11

Results for zero‐inflated negative binomial models showed that the three best models explained pair habitat use within ΔAIC < 2 (Table 2). The base model (SHORE + FIELD; these variables were included in all models) had the lowest AIC value. The null model (intercept only) had the poorest fit. Pair numbers at the water bodies increased with shoreline length and field percentage, and ponds had fewer pairs than lakes. Food index appeared not to contribute (Table 3).

**TABLE 2:** Models explaining the number of pairs and broods at the water bodies. Only best models with ΔAIC < 2, where Δ = AIC_*i*_ − AIC_min_, are shown.

Model	df	AIC	ΔAIC	*w*
Pairs				
SHORE + FIELD	6	322.886	0.000	0.424
TYPE + SHORE + FIELD	7	323.645	0.759	0.290
FOOD + SHORE + FIELD	7	324.850	1.964	0.159
Intercept only	1	348.806	25.920	0.000
Broods				
FOOD + PAIRS + FIELD	7	178.883	0.000	0.656
FOOD + TYPE + PAIRS + FIELD	8	180.331	1.448	0.318
Intercept only	1	192.818	13.935	0.000

*Note*: TYPE = lake or pond, SHORE = water body shoreline (km), FOOD = invertebrate food index, FIELD = field percentage within 1000 m buffer zone around the water body.

**TABLE 3:** Model averaged parameter estimates, their standard errors and unconditional 95% confidence intervals from models explaining habitat use of pairs and broods.

	Estimate	SE	95% CI	
			Lower	Upper
Pairs				
FOOD	0.013	0.007	−0.001	0.028
TYPE (pond)	−0.490	0.038	−0.579	−0.429
SHORE	0.297	0.022	0.247	0.336
FIELD	0.028	0.000	0.026	0.029
Broods				
FOOD	0.144	0.006	0.132	0.158
TYPE (pond)	−0.331	0.365	−0.719	0.874
PAIRS	0.066	0.012	0.058	0.105
FIELD	0.006	0.001	0.005	0.011

*Note*: Estimates are based on all models in the candidate model set using Akaike weights as weighting factors. TYPE = pond (lake represented by intercept), SHORE = water body shoreline (km), FOOD = invertebrate food index, FIELD = field percentage within 1000 m buffer zone around the water body.

Abbreviation: CI, confidence interval.

Two well‐fitting brood models were within ΔAIC < 2 and both included ‘FOOD’ (Table 2). The null model (intercept only) had again the poorest fit. Brood numbers increased with food abundance (Table 3), but less so with number of pairs. Field percentage had only a weakly positive but significant effect on brood numbers.

### 3.2 Nest survival

**FIGURE 2:**
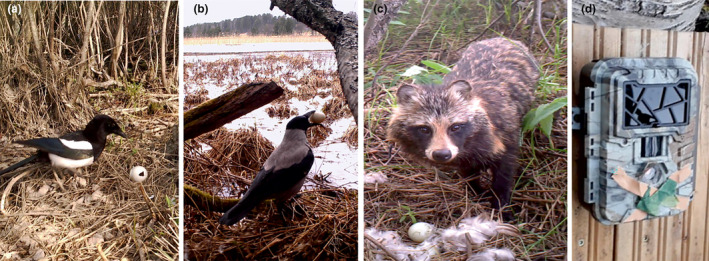
Camera trapping revealed that the most common nest predator species were (a) Eurasian magpie (photo by Niteforce Professional Trail Camera 12 MP), (b) hooded crow (photo by Uovision UV595‐Full HD 12 MP) and (c) raccoon dog (still from video by Uovision with +2 eyeglass lens: video taken after the actual experiments, lenses were not used in the actual experiments). (d) To adjust the focus of wildlife cameras to less than 1 m, we attached +2 eyeglass lens (‘backwards’) in front of the wildlife camera lens. Tape was then camouflaged.

Of the artificial nests, 44% (*N* = 39) were depredated in 2017 and 39% (*N* = 35) in 2018. The 2‐year average nest predation rate at Evo was 24% (*N* = 11 and *N* = 12, respectively) and at Maaninka 61% (*N* = 27 and *N* = 27, respectively). Camera trapping revealed that most common nest predator species were Eurasian magpie (*Pica pica*, *N* = 25, 33% of the depredated nests), raccoon dog (*Nyctereutes procyonoides*, *N* = 12, 16%), hooded crow (*Corvus corone cornix*, *N* = 12, 16%; Figure 2) and Eurasian jay (*Garrulus glandarius*, *N* = 8, 11%). To a lesser extent, nests were destroyed by pine martens (*Martes martes*, *N* = 5, 7%) and common ravens (*Corvus corax*, *N* = 5, 7%), while one nest per species was predated by the American mink (*Neovison vison*), western marsh harrier (*Circus aeruginosus*), common crane (*Crus crus*), European badger (*Meles meles*) and domestic dog (*Canis lupus familiaris*). Two nest predators remained unknown due to camera failure (one camera had a full memory card and the other camera for an unknown reason had not reacted to the predator). An average depredation time for the artificial nests was 2.8 days (standard deviation 1.7). Only four nests were depredated in less than 5 h after establishment (2 in 2017 and 2 in 2018; three times by hooded crow and one time by Eurasian magpie, minimum time 1.5 h), suggesting that the predators did not follow researchers to the nests.

**FIGURE 3:**
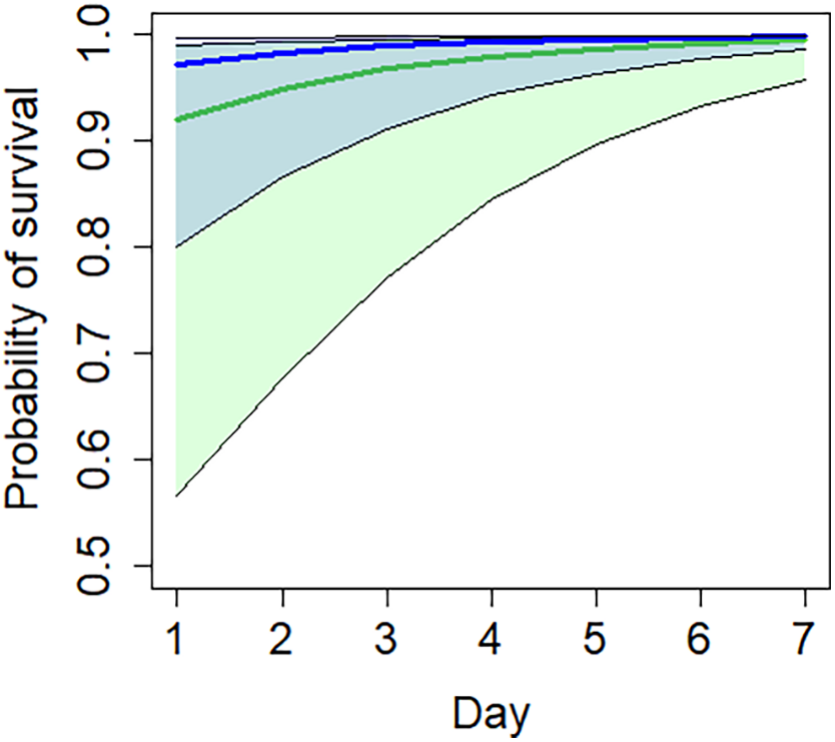
Daily nest survival rate of artificial nests at permanent lake shore (green) and pond shores (blue) with 95% confidence intervals during the 7‐day experiments in 2017–2018 (see parameters in Table 5).

Daily nest survival was higher in forest compared to shoreline (Table 4). Nest survival also tended to have a slight but significant negative relationship with field percentage around the water bodies. As expected, shoreline nest daily survival was higher around ponds than around lakes (Table 5, Figure 3). Again, nest survival tended to have a slight but significant negative relationship with field percentage around the water bodies.

**TABLE 4:** Model estimate for the daily survival rate of artificial nests on shoreline and forest.

	Estimate	SE	*z*‐Value	*p*
(Intercept)	2.182	0.373	5.856	<.001
DATE	0.512	0.077	6.671	<.001
HABITAT (Forest)	0.708	0.266	2.661	.008
FIELD	−0.021	0.006	−3.719	<.001

*Note*: DATE = exposure day (1…7), HABITAT = forest (categorical factor, shoreline represented by intercept), FIELD = field percentage within 1000 m buffer zone around the water body. Random effect standard deviation for NESTPAIR_ID = 0.69.

**TABLE 5:** Model estimate for the daily survival rate of artificial nests on lake and pond shoreline.

	Estimate	SE	*z*‐Value	*p*
(Intercept)	1.983	0.603	3.286	.001
DATE	0.473	0.125	3.783	<.001
TYPE (Pond)	1.122	0.534	2.102	.036
FIELD	−0.029	0.009	−3.089	.002

*Note*: DATE = exposure day (1…7), TYPE = pond (categorical factor, lake represented by intercept), FIELD = field percentage within 1000 m buffer zone around the water body. Random effect standard deviation for NESTPAIR_ID = 1.12.

### 3.3 Invertebrate food abundance

**FIGURE 4:**
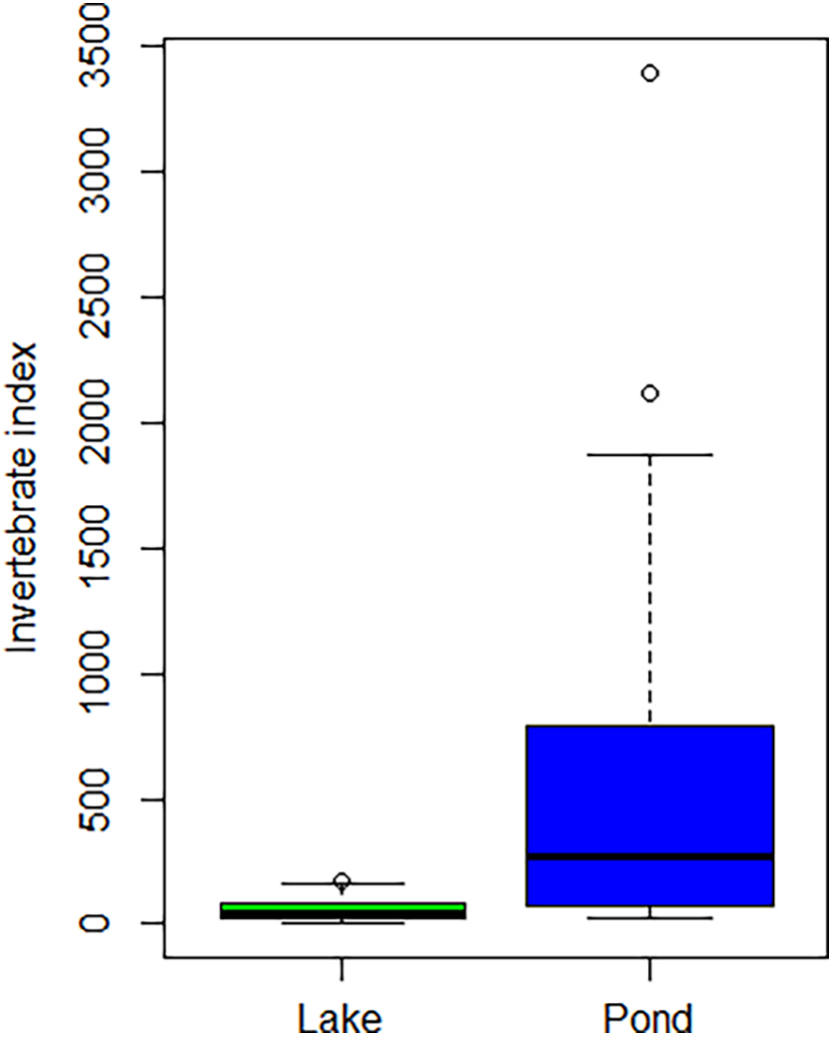
Invertebrate food abundance (invertebrate index) in permanent lakes and ponds during the study in 2017–2018. Box plot shows the median, interquartile range and whiskers indicate the range. Circles indicate outliers.

Water body type affected the invertebrate food index. Ponds had a higher index than permanent lakes (Table 6, Figure 4), indicating that ponds are more food‐rich habitats than lakes. Shoreline length or field percentage around the water body did not explain the invertebrate index.

**TABLE 6:** Parameters of the model explaining invertebrate food index.

	Estimate	SE	*t*‐value	*p*‐Value
Intercept	0.671	1.424	0.471	.640
TYPE (pond)	3.618	1.604	2.256	.030
SHORE	−0.284	0.512	−0.554	.582
FIELD	0.036	0.026	1.408	.166

*Note*: TYPE = pond (lake represented by intercept), SHORE = water body shoreline (km), FIELD = field percentage within 1000 m buffer zone around the water body. Random effect standard deviation for WATER BODY_ID = 3.373.

## 4 DISCUSSION

Our results indicate that duck densities were higher in the agricultural landscape. However, ducks appear to face a potential trade‐off, because the agricultural landscape also had a higher nest predation rate than the forest landscape as revealed by experimental nests equipped with wildlife cameras (see also Gunnarsson & Elmberg, 2008). Brood production per pair probably reflects this trade‐off: production was higher in the forest landscape than in the agricultural landscape, which suggests the occurrence of a high nest predation rate and/or high brood mortality in the agricultural landscape. The results imply that the predator detection at artificial duck nests with camera trapping could actually reflect their visitation to natural nests. However, our results only concern the early egg‐laying period, while predation later in the nest period, during incubation, likely incurs a higher cost to the female (Ackerman et al., 2003; Dyson et al., 2020).

Interestingly, the corresponding trade‐off between food abundance and potential nest predation risk was not evident at the habitat level, because ponds rich in food also have low nest predation rates. The camera trapping data show that the nests on the shores of seasonal, beaver or man‐made ponds had higher survival than nests on the shoreline of permanent lakes. Because we tried to keep the nest cover constant between the experimental nests, this difference in survival rates probably arises from more heterogeneous shoreline habitats of ponds and/or the availability of other abundant food resources ponds offer for predators. It has been suggested that nest survival is a combination of large‐scale environmental factors and local nest‐site characteristics. Landscape productivity can affect general predator and prey abundance, but at the nest‐site level vegetation and nest location might affect nest detectability and predator behaviour (Ringelman et al., 2018). For example, predator foraging in the landscape may be concentrated at habitat edges (Andrén, 1995), such as the interface between terrestrial and aquatic habitats. The occurrence of the edge effect may depend on the predator community and predator behaviour (Pasitschniak‐Arts et al., 1998), and for instance, whether productive wetlands attract and support high number of predators (Stephens et al., 2005).

In the Evo area, mammalian predators have been found to occur more often around beaver ponds than permanent lakes (Nummi, Liao, et al., 2019). Still, higher predator occurrence around the beaver ponds was not reflected in the nest predation results, indicating that indeed some habitat‐related factors are working in favour of higher nest survival. It is possible that variability in the shoreline creates circumstances under which predators probably are not able to form long‐term search images, i.e. circumstances resembling those considered by Nams (1997) for prey aggregated in space or time (see also Ellis et al., 2020). We suggest that because predators may use spatial memory to improve searching efficiency (Phillips et al., 2004), their search around permanent lakes is more regular and effective than around temporally unpredictable ponds. This underlines the potential importance of seasonally flooded ponds for breeding ducks, especially in agricultural areas.

Overall, nest predation risk was lower in forests compared to shoreline nests, indicating the occurrence of the edge effect between terrestrial and aquatic ecotones. Several currently threatened and endangered duck species in Finland are typically nesting on the ground near the shoreline (e.g. common pochard *Aythya ferina*, tufted duck) and may suffer from stronger nest predation rates than more flexible nesters (e.g. mallard and teal; e.g. Holopainen et al., 2024; Pöysä et al., 2019, 2023). Nesting in forests may be safer, but on the other hand, newly hatched ducklings will have to move a long way in water and face potentially higher mortality risk in the inhospitable matrix (Pöysä & Paasivaara, 2006).

Increased predator abundance and diversity are typical, especially for fragmented landscapes (Andrén, 1995; Pasitschniak‐Arts & Messier, 1995). The pattern is particularly pronounced in agricultural landscapes, where there are already high numbers of predators, such as corvids (Andrén, 1992; Holopainen et al., 2020a; Roos, 2002). The results support these observations: nest predation risk was higher in the agricultural landscape, where high rates of corvid predation and richer predator communities were observed with similarly executed camera trap‐artificial nest experiments (Holopainen et al., 2020a, 2020b). Camera trap studies conducted both with artificial nests (Holopainen et al., 2020b) and natural nests (Bell & Conover, 2023) have proven that after the initial depredation event, disturbed nests are often visited by multiple secondary predators. Multiple mammalian visits lead not only to an increased egg depredation rate but also a higher mortality risk for the incubating female. Indeed, hens often abandon (partially) depredated nests and even if incubation is continued, hatching success rate is low (Ackerman et al., 2003; Bell & Conover, 2023).

In Europe, the overall predator populations have increased during the last decades threatening bird populations (Roos et al., 2018). In addition to native species, invasive alien predators such as raccoon dogs have dispersed widely and threaten native bird species (Bonesi & Palazon, 2007; Jauni et al., 2021; Kauhala & Kowalczyk, 2011). Raccoon dog nest predation can be destructive on islands (Dahl & Åhlén, 2018), but its role as mainland duck nest predator has remained unclear (Kauhala, 2004; Kauhala & Auniola, 2001; Nummi, Väänänen, et al., 2019; Sidorovich et al., 2008). Corvids and raccoon dog have been found to be responsible for most of the nest depredation occurring at experimental nests mimicking the situation in the early stage of egg laying (Holopainen et al., 2020a). Without camera traps, predator identification is uncertain as it relies on the remains of eggshells or other cues on the nest site (Larivière, 1999).

We recognize that artificial nests give an uncertain reflection of actual nest predation, and thus the intention in this study was not to evaluate actual predation rates but only to study the habitat‐specific relative predation risk. Many important differences (e.g. different predator species) exist between real and artificial nests that decrease the correspondence and are thus recommended to consider whenever conducting artificial nest experiments (Butler & Rotella, 1998; Pärt & Wretenberg, 2002; Richardson et al., 2009; Whelan et al., 1994; Wilson & Brittingham, 1998). The predator species we observed in the camera pictures are known predators of real duck and other ground‐nesting game birds' eggs (Kauhala & Ihalainen, 2014; Møller, 1983; Opermanis et al., 2001; Pöysä et al., 1997), and therefore we assume that the observed species do not differ from the actual nest predator assemblage. As Anthony et al. (2006) showed with dusky Canada geese (*Branta canadensis occidentalis*) artificial nests can be used to identify the potential nest predator species and that the predator species ratios can correspond to those of the real nests. Our artificial nest density was low ensuring that observations were independent. The lacking hen problem was avoided by focusing only on the early egg‐laying stage when females are not on their nests, so the setup resembles the actual situation; the presence of females might attract different predators to the nest (Dyson et al., 2020). We also acknowledge that this study design potentially emphasizes the role of visual predators, such as corvids, as nests were not necessarily hidden as efficiently as a duck hen's nest would be. High corvid predation rates may also be expected to occur at the early real nests, as the duck nest predation rate in North America during the early part of the breeding season was observed to positively relate to American crow (*Corvus brachyrhynchos*) activity (Johnson et al., 1989).

The correspondence of the artificial nests with actual nest success cannot be assessed. While there are still uncertainties in this method, we emphasize that the problems underlined by the earlier studies have been considered and the differences between real and artificial nests were accordingly minimized; thus, we suggest that our data are suitable for detecting trends in predation rates in relation to habitat (Wilson & Brittingham, 1998).

As expected, ponds (seasonal, beaver and man‐made) were more invertebrate‐rich habitats than permanent lakes, while contrary to the hypothesis, the percentage of field land around the water bodies did not influence the invertebrate index. Selecting a pond instead of a lake as a breeding habitat would thus simultaneously minimize nest predation risk and maximize food availability in any landscape.

Habitat use of duck pairs was not associated with invertebrate food, whereas duck broods preferred habitats richer in food. The number of broods at the water bodies was only weakly dependent on the number of pairs, which can be a reflection of differing habitat requirements of pairs and broods (Holopainen et al., 2015) or high nest predation and brood mortality. Sjöberg et al. (2000) showed for mallards that all lakes used by pairs are not suitable for broods, the difference in lake use between pairs and broods being due to food limitation at the brood stage (Gunnarsson et al., 2004). In boreal lakes food limitation can be intensified due to food competition between ducks and fish (Nummi et al., 2016). Income breeders, like teal, seem to avoid brood‐stage food limitation by congregating in beaver ponds and seasonal ponds where invertebrate production is high and the habitat structure favourable for brood foraging (Nummi & Hahtola, 2008; Nummi & Holopainen, 2014).

Interestingly, the results did not show that duck pairs or broods used ponds more than permanent lakes. It is possible that ducks visit food‐rich ponds for foraging in very short periods, reducing the ability to detect them there (Nummi, Suontakanen, et al., 2019). Waterbird species may also differ in their ability to respond to environmental factors, such as habitat variability (Nummi & Pöysä, 1997; Wiens, 1976). In Evo it is known that teal brood production is following the flood dynamics created by the beaver and spring floods (Holopainen et al., 2014). Accordingly, lapwings (*Vanellus vanellus*) are known to nest in higher densities around flooded footdrains, and chicks forage on the wet mud around these wet features supporting invertebrate‐rich habitats (Eglington et al., 2008, 2010).

### 4.1 Conservation implications

Successful management of ducks would demand an understanding of the relationship between habitat availability and predation pressure (Drever et al., 2004). This study emphasizes the benefits of the availability of different water body types for breeding ducks. We showed that flooded and/or seasonal ponds might be especially good habitats, where two important limiting factors of the breeding season – nest survival and amount of invertebrate food – are higher there than on permanent lakes.

Kubelka et al. (2018) showed that shorebirds have experienced a worldwide increase in nest predation over the past decades and that the pattern is especially pronounced in the high northern latitudes. Twelve of the 19 duck species living in Finland are already classified as threatened to some degree by the Finnish red list (Lehikoinen et al., 2019), underlining the urgent need for conservation actions. Our results indicate that while duck pair and brood densities are higher in an agricultural landscape, brood production seems to be higher in forested landscapes with lower nest predation rates. Therefore, predator management especially in agricultural landscapes could enhance nest survival there. Our results are in line with other studies suggesting that duck species nesting at eutrophic lakes in agricultural areas and preferring especially shorelines as nesting places may suffer from high nest predation rates, which may contribute to the declining population trends (Holopainen et al., 2024; Jauni et al., 2021; Lehikoinen et al., 2016; Pöysä & Linkola, 2021). It is suggested by several studies that the nest predation pressure around these lakes has increased due to the appearance of alien predators (Holopainen et al., 2021; Nummi, Väänänen, et al., 2019; Pöysä & Linkola, 2021). Controlling predators, especially alien species, would thus be an important conservation action to improve duck breeding success (Dahl & Åhlén, 2018; Garrettson & Rohwer, 2001; Jaatinen et al., 2022).

Considering that flooded and seasonal ponds appeared to be especially good habitats in terms of nest survival and food abundance, much more effort should be put into their conservation. In general, seasonal pond ecosystems in the boreal biome remain poorly studied, even so that for example in Finland the habitat type does not have a conservation status evaluation done due to the lack of information (Lammi et al., 2018). The loss of seasonal ponds has been dramatic in boreal biome, including Finland (Kuusisto et al., 1998), due to drainage, destruction and water regulation (Colburn, 2004). Furthermore, it is predicted that climate change will reduce the extent of snowmelt‐dependant spring flooding in the future (Veijalainen et al., 2010), decreasing further the abundance of seasonal ponds. In addition to wetland restoration and blocking up drains, the lack of flooded waters may be mitigated by managing beavers (Hood & Bayley, 2008; Nummi & Holopainen, 2020) or creating man‐made wetlands (Čehovská et al., 2022; Danell & Sjöberg, 1982; Eglington et al., 2008).

## AUTHOR CONTRIBUTIONS

**Sari Holopainen:** Conceptualization (lead); data curation (lead); formal analysis (lead); funding acquisition (lead); investigation (lead); methodology (lead); project administration (lead); resources (lead); software (lead); supervision (lead); validation (lead); visualization (lead); writing – original draft (lead); writing – review and editing (lead). **Elmo Miettinen:** Conceptualization (supporting); data curation (supporting); formal analysis (supporting); funding acquisition (supporting); investigation (supporting); methodology (supporting); project administration (supporting); resources (supporting); software (supporting); supervision (supporting); validation (supporting); visualization (equal); writing – original draft (equal); writing – review and editing (supporting). **Veli‐Matti Väänänen:** Conceptualization (lead); data curation (equal); formal analysis (supporting); funding acquisition (supporting); investigation (equal); methodology (equal); project administration (supporting); resources (equal); software (equal); supervision (equal); validation (equal); visualization (supporting); writing – original draft (equal); writing – review and editing (equal). **Petri Nummi:** Conceptualization (lead); data curation (equal); formal analysis (supporting); funding acquisition (supporting); investigation (supporting); methodology (supporting); project administration (supporting); resources (equal); software (supporting); supervision (supporting); validation (supporting); visualization (supporting); writing – original draft (equal); writing – review and editing (equal). **Hannu Pöysä:** Conceptualization (equal); data curation (supporting); formal analysis (supporting); funding acquisition (supporting); investigation (supporting); methodology (equal); project administration (supporting); resources (equal); software (supporting); supervision (supporting); validation (equal); visualization (supporting); writing – original draft (equal); writing – review and editing (equal).

## FUNDING INFORMATION

Maj and Tor Nessling Foundation and Haavikko‐foundation funded the work of SH and Suomen Riistanhoito‐Säätiö and Lammi Biological Station funded the work of EM. Kuopion Luonnonystävät gave a grant for wildlife cameras for V‐MV.

## CONFLICT OF INTEREST STATEMENT

We declare that the authors of this article have no conflicts of interest.

## Supporting information


Appendix S1

## Data Availability

We confirm that the entire database used in this article is available in the Supporting Information of this manuscript.
